# Curcumin Protects SDH2 Mutant from Oxidative Stress and Improves Mitochondrial Function: Application Potential for Complex II Deficiency

**DOI:** 10.3390/ijms27125253

**Published:** 2026-06-10

**Authors:** Yi Liu, Na Wang, Heng Cai

**Affiliations:** College of Biotechnology and Pharmaceutical Engineering, Nanjing Tech University, Nanjing 211816, China; llm@njtech.edu.cn (Y.L.); wangna@njtech.edu.cn (N.W.)

**Keywords:** complex II deficiency, mitochondrial disorders, oxidative stress, curcumin

## Abstract

Complex II deficiency is a rare inherited mitochondrial disorder characterized by structural and functional deficiency of complex II, for which there is currently no definitive drug treatment. Curcumin is a polyphenolic compound with antioxidant, anti-inflammatory, anti-tumor, and anti-aging properties. By constructing a mutant strain of the *Saccharomyces cerevisiae* *SDH2* gene, we can mimic the functional defects of Complex II caused by human *SDHB* mutations, and then explore the ameliorative effect of curcumin on Complex II functional defects. Cell viability was assessed using MTT and CFU. Antioxidant capacity was evaluated by measuring DCFH-DA and antioxidant enzyme activity, while the expression levels of respiratory chain-related genes were detected by qRT-PCR. Experimental results demonstrate that curcumin can restore cell growth and viability, scavenge ROS from cells as well as positively regulate mitochondrial function; however, the above results are regulated by the concentration of curcumin. In conclusion, these findings provide experimental support for curcumin as a preliminary intervention for Complex II deficiency and other mitochondrial diseases, further enriching the evidence for the potential application of curcumin in mitochondrial-related diseases.

## 1. Introduction

Complex II deficiency is a rare autosomal recessive neurometabolic disorder. Complex II, encoded by nuclear genes, participates in both the tricarboxylic acid cycle and the mitochondrial electron transport chain; thus, its dysfunction severely impairs cellular energy metabolism [1]. This leads to reduced ATP production, cellular damage, and cell death [2]. The clinical manifestations of this disease are highly heterogeneous, ranging from severe multisystem involvement in infantile forms to isolated myopathy with adult onset [3], Common symptoms include white matter encephalopathy, optic atrophy, cardiomyopathy, developmental delay, and ataxia. Some patients may progress to Leigh syndrome—a severe encephalopathy characterized by progressive neurodegeneration, developmental delay, and dystonia [4]. It is estimated that complex II deficiency accounts for approximately 2–7% of all Leigh syndrome cases [5].

Treatment of mitochondrial diseases is usually based on vitamin supplements, dietary modifications, and exercise [6], and a variety of pharmacological treatments are currently being investigated to assess their potential as therapeutic treatments for inherited mitochondrial diseases. There are a number of treatments with considerable potential for development, such as Idebenone, a synthetic quinone analog of coenzyme Q10 that may have the potential to restore cellular ATP production, which also acts as an antioxidant by protecting lipid membranes and mitochondria from oxidative damage [7]. It has shown improvement in indicators in trials treating patients with LHON [8].

Curcumin is a natural dietary polyphenol extracted from turmeric with different biological and pharmacological properties including antioxidant [9], immunomodulatory, anti-inflammatory, antitumor, antirheumatic, and anti-aging [10]. Curcumin’s antioxidant activity is dictated by its chemical structure, with both phenolic hydroxyl (OH) and β-diketone moieties contributing to free radical neutralization, which varies among different structures and scavenging capacity for different types of free radicals [11]. Notably, mitochondria represent a key target for curcumin-mediated protective effects. Curcumin regulates mitochondrial function through multiple pathways: in terms of mitochondrial energy metabolism, curcumin inhibits TORC1 activity, enhances ATP synthesis, and improves the survival of cells with mitochondrial functional defects by inducing moderate oxidative stress to activate cellular energy stress responses [12]. Regarding mitochondrial antioxidant defense, curcumin directly scavenges mitochondrial ROS, induces endogenous antioxidant enzymes via the Nrf2 pathway, and reduces oxidative damage markers such as MDA and protein carbonyls [13], reflecting its multi-dimensional protective effects against mitochondrial oxidative injury [14]. In terms of mitochondrial apoptosis regulation, curcumin increases the level of anti-apoptotic Bcl-2 protein in mitochondria, reduces the translocation of cytochrome c to the cytosol, impairs caspase activation, and consequently reduces neuronal cell death [15].

Various model systems have been established to investigate the pathological mechanisms and potential therapeutic strategies for complex II deficiency. In animal models, Frances Fan and colleagues explored the therapeutic potential of rapamycin for SDH deficiency using a Drosophila model, providing important insights for interventional studies on complex II deficiency [16]. Compared with animal models such as Drosophila, yeast cells offer distinct advantages, including a short culture period, low experimental cost, facile and rapid genetic manipulation, and relatively well-defined metabolic pathways [17]. Furthermore, the sophisticated application of high-throughput screening techniques enables efficient assessment of thousands of phenotypic traits in *Saccharomyces cerevisiae* [17]. In mitochondrial SDH disease research, yeast models have demonstrated significant value. For instance, Panizza et al. [18] systematically evaluated the pathogenicity of SDHB, SDHC, and SDHD missense mutations using a yeast model, demonstrating that yeast is an excellent functional model for validating SDHB pathogenicity. Although previous studies have employed yeast models to assess the pathogenicity of SDH mutations, no study to date has systematically investigated the protective effects or underlying mechanisms of natural products on SDH-deficient yeast cells.

In this work, we screened two clinically reported SDHB missense mutation sites (Asp48Val [19] and Ala102Thr [5]), both of which are directly associated with the development of complex II deficiency. Given the current lack of targeted therapeutic strategies for complex II deficiency, and considering that curcumin, as a natural dietary polyphenol, exerts multi-target mitochondrial regulatory effects, we aimed to investigate the therapeutic potential of curcumin for this disease. We used *Saccharomyces cerevisiae* to mimic the mutation sites introduced into complex II deficiency by mutating Asn-42 to Val and Ala-96 to Thr in the yeast *SDH2* gene.

We used exogenous H_2_O_2_ to simulate the oxidative stress microenvironment in SDHB-deficient cells during aging or late-stage disease. Compared with superoxide inducers like paraquat or menadione (which primarily target complex I), H_2_O_2_ better reflects the late-stage oxidative stress in complex II deficiency, where endogenous $O_{2}^{-}$ is dismutated by SOD to H_2_O_2_—a stable, membrane-permeable molecule that mediates extensive oxidative damage [20]. Additionally, yeast SDH2 mutants are highly sensitive to H_2_O_2_ stress, and this model has been widely used in neurodegenerative disease research, demonstrating that H_2_O_2_ effectively reveals phenotypic consequences of complex II dysfunction [21].

Based on the above model, we used hydrogen peroxide treatment was used to simulate the state of increased oxidative stress in senescent cells or late-stage diseases. By analyzing the phenotypes of mutant strains under treatment with different concentrations of curcumin, we evaluated the impact of curcumin on physiological indicators associated with complex II functional deficiency. For example, we examined whether curcumin improves stress tolerance, enhances cell viability, and reduces oxidative stress in mutant strains, thereby providing experimental evidence for its potential application in the intervention of relevant human diseases. Utilizing *Saccharomyces cerevisiae* in-depth research on the mechanism of curcumin’s influence on cell physiological processes will provide a basis for further revealing the mechanism of curcumin’s action in higher organisms.

## 2. Results

### 2.1. Screening and Validation of Pathogenic Mutations in the SDHB Gene

**Figure 1:**
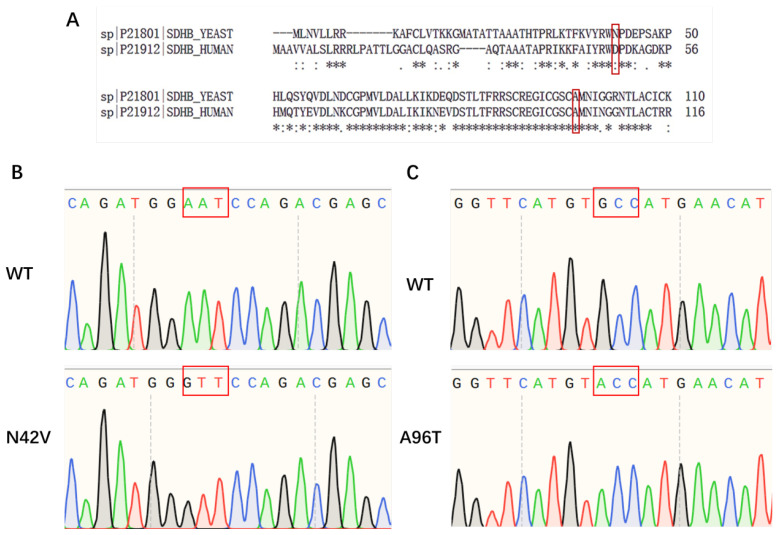
Screening and validation of pathogenic mutations in the *SDHB* gene. (**A**) Sequence comparison of the yeast *SDH2* gene with the human *SDHB* gene. Sequence alignment symbols: * identical residues; : highly conserved substitutions; . semi-conserved substitutions. (**B**,**C**) Sequencing profiles of the yeast *SDH2* wild-type gene with N42V and A96T mutantstrains. Red boxes indicate the mutation sites: (**B**) N42V (AAT → GTT, Asn → Val); (**C**) A96T (GCC → ACC, Ala → Thr).

We selected two clinically reported missense mutations (Asp48Val and Ala102Thr) associated with complex II deficiency. The Asp48Val mutation originated from a patient described in the literature [19], who presented with abnormal developmental progression, motor dysfunction, characteristic imaging findings, and metabolic abnormalities. Histochemical and spectrophotometric analyses confirmed a severe and isolated complex II deficiency in this patient. Further genetic analysis revealed a novel homozygous variant in exon 2 of the *SDH2* gene, resulting in the substitution of aspartic acid (Asp) by valine (Val) at position 48 of the SDHB subunit. Sequence alignment revealed that although the amino acid at position 48 of human SDHB and that at position 42 of yeast SDH2 are not identical, both are polar amino acids (Figure 1A).

The Ala102Thr mutation originated from another proband reported in the literature [5], who presented with febrile seizures, neurodegeneration, optic atrophy, and elevated lactate levels in both plasma and cerebrospinal fluid. Whole-exome sequencing identified a homozygous c.304G>A (Ala102Thr) variant in the *SDH2* gene, with both parents being heterozygous carriers, consistent with an autosomal recessive inheritance pattern. In silico analyses predicted this variant to be deleterious. Sequence alignment revealed that alanine at position 102 of human SDHB is conserved with alanine at position 96 of yeast SDH2 (Figure 1A).

To investigate the impact of these mutations on complex II function and to further evaluate, at the molecular level, the ameliorative effect of curcumin on complex II functional defects induced by pathogenic mutations, this study used *Saccharomyces cerevisiae* BY4741 as the background strain. First, the *SDH2* gene was knocked out via homologous recombination to generate the BY4741Δ*sdh2* deletion strain. Subsequently, the wild-type *SDH2* gene was reintroduced into this deletion strain to construct the complemented strain Δ*sdh2(+)*. Meanwhile, the Asp48Val and Ala102Thr mutations of human SDHB were correspondingly introduced into the yeast *SDH2* gene to generate the N42V and A96T mutant strains, respectively. Sequencing results confirmed the successful introduction of the target mutations (Figure 1B,C).

### 2.2. Cellular Characterization of Mutant Strains

**Figure 2:**
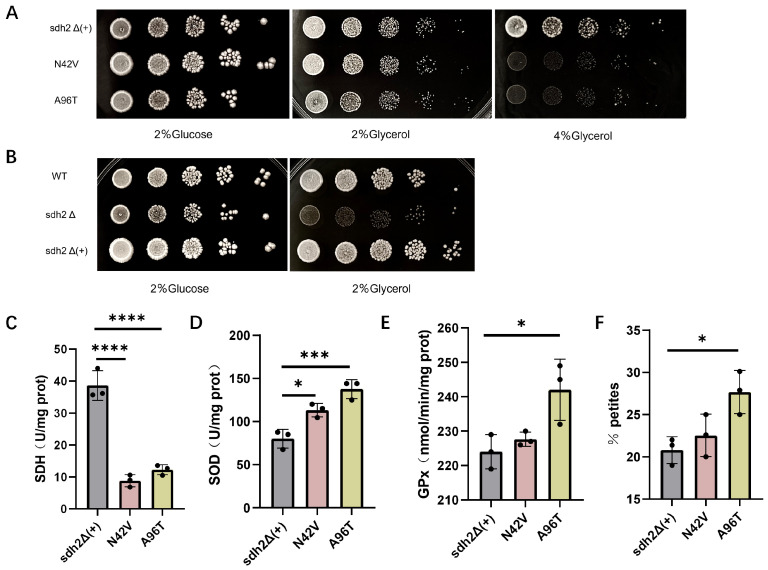
Cellular characterization of mutant strains. (**A**,**B**) Oxidative growth phenotype; (**C**) succinate dehydrogenase (SDH) activity in *sdh2*Δ(+) and mutant strains N42V and A96T; (**D**) superoxide dismutase (SOD) activity in *sdh2*Δ(+) and mutant strains N42V and A96T; (**E**) Glutathione peroxidase (GPx) activity assay of *sdh2*Δ(+) and mutant strains N42V and A96T; (**F**) percentage of small colonies in *sdh2*Δ(+) and mutant strains N42V and A96T. Enzyme activities were normalized to total protein concentration and expressed as follows: SDH activity (**C**) in U/mg protein, SOD activity (**E**) in U/mg protein, and GPx activity (**F**) in nmol/min/mg protein. All data are presented as mean ± SD from three independent experiments. Statistical analyses were performed using one-way ANOVA followed by Tukey’s post hoc test. * *p* < 0.05, *** *p* < 0.001, **** *p* < 0.0001.

In glycerol medium, yeast strains are unable to depend on fermentative metabolic pathways for energy supply and instead produce energy mainly through the mitochondrial oxidative phosphorylation pathway, which is dependent on the involvement of functionally intact respiratory chain complexes. The *sdh2*Δ strain exhibited markedly impaired growth in glycerol medium, while the introduction of the pYX212 plasmid containing the SDH2 coding sequence rescued its growth ability under non-fermentable carbon source conditions (Figure 2A). This finding verifies that the *SDH2* gene is capable of normal expression and can perform its biological functions in the complemented strain. Assessing the growth of mutant strains on fermentable and non-fermentable carbon sources is a fundamental and rapid approach to identify pathogenic mutations. The *sdh2*Δ strain and the mutant strains grew normally in glucose-based medium, yet growth was markedly inhibited in glycerol medium (Figure 2A), indicating a defect in mitochondrial respiratory function in the tested strains. Consistent with this, succinate dehydrogenase (SDH) activity was significantly reduced in mutant strains (Figure 2C), indicating a disruption of normal SDH function that likely impacts cellular energy metabolism.

The presence of small colonies—characterized by slow growth and reduced size—often reflects mtDNA mutations or deletions that compromise respiratory chain integrity. Given that mtDNA lacks histone protection and resides in close proximity to the mitochondrial respiratory chain, it is highly susceptible to ROS attacks. Oxidatively damaged mtDNA tends to accumulate replication errors [22], leading to base mismatches and subsequent micro-mutations [23]. Indeed, the frequency of micro-mutations was elevated in the mutant strains (Figure 2F), suggesting increased ROS production and consequent oxidative damage to mtDNA.

Superoxide dismutase (SOD) protects cells by scavenging superoxide anions. The SOD levels in mutant strains were significantly elevated (Figure 2D), suggesting enhanced production of superoxide anions ($O_{2}^{-}$) in these strains, thereby necessitating increased SOD expression to mitigate oxidative stress. Glutathione peroxidase (GPx), an essential peroxidase enzyme that utilizes reduced glutathione (GSH) as a substrate, catalyzes the reduction of toxic hydrogen peroxide (H_2_O_2_) into harmless hydroxyl compounds, thus protecting cells from ROS-induced damage. Measurement of GPx activity provides an index of the cellular capacity to detoxify H_2_O_2_. In mutant strains, GPx activity was significantly upregulated (Figure 2E), indicating elevated intracellular ROS levels and a compensatory cellular response to enhance H_2_O_2_ clearance, thereby contributing to the maintenance of redox homeostasis.

### 2.3. Curcumin Restores Cell Growth and Vitality

**Figure 3:**
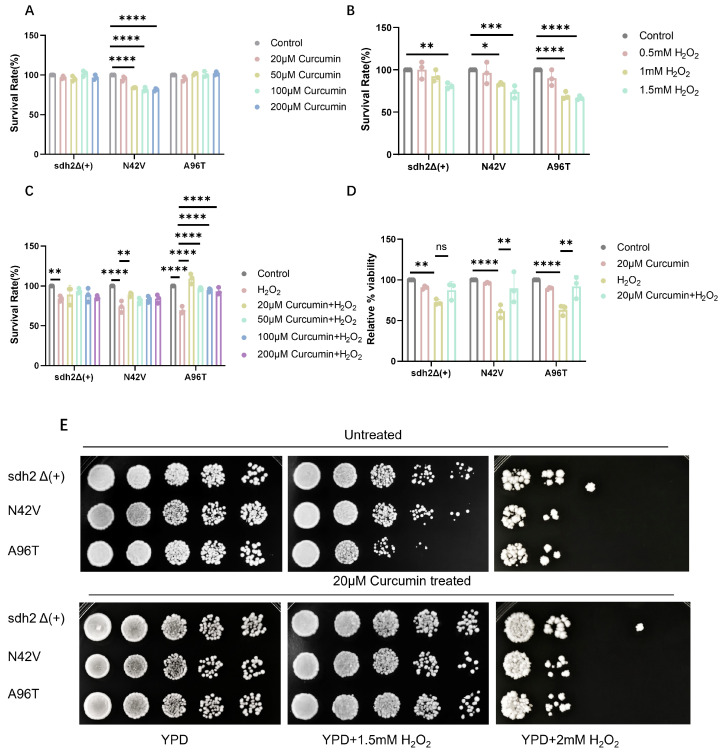
Curcumin restores cell growth and vitality. (**A**–**C**) MTT assay to determine cell viability under curcumin and hydrogen peroxide (H_2_O_2_) treatment; (**D**) colony-forming unit (CFU) assay to determine cell viability; Data are presented as percentages relative to the untreated control of the same genotype (set as 100%), reflecting the relative metabolic activity of each treatment group compared to the control. (**E**) *sdh2*Δ(+) and mutant strains N42V and A96T after curcumin treatment in solid YPD medium containing different concentrations of H_2_O_2_. All data are presented as mean ± SD from three independent experiments. One-way ANOVA was performed to compare each treatment group (various concentrations of curcumin and/or H_2_O_2_) with its own untreated control. Tukey’s post hoc test was used for multiple comparisons with correction. ns, not significant, * *p* < 0.05, ** *p* < 0.01, *** *p* < 0.001, **** *p* < 0.0001.

To investigate the effects of curcumin and H_2_O_2_ on the cell viability of SDH2 mutant strains, curcumin and H_2_O_2_ were individually applied to the strains, and cell viability was assessed using the MTT assay. In studies on yeast models of mitochondrial dysfunction, curcumin has been shown to exert concentration-dependent regulatory effects on the lifespan of defective cells within the range of 12.5–200 μM [12]. Based on these findings, four concentrations—20, 50, 100, and 200 μM—were selected for concentration gradient experiments in this study. The results showed that none of the four curcumin concentrations had a significant effect on the cell viability of the Δ*sdh2(+)* strain or the A96T mutant strain. However, treatment with 50, 100, and 200 μM curcumin led to a marked decrease in the cell viability of the N42V mutant strain (Figure 3A), indicating that the N42V mutant is more sensitive to high-concentration curcumin-induced stress.

To verify the effects of different concentrations of H_2_O_2_ on cell viability, three concentrations were tested in this study. As shown in Figure 3B, treatment with 1.5 mM H_2_O_2_ significantly reduced the cell viability of both the Δ*sdh2(+)* strain and all mutant strains. Therefore, 1.5 mM H_2_O_2_ was selected as the uniform concentration for subsequent experiments. Next, we investigated whether four different concentrations of curcumin could alleviate the cellular damage induced by 1.5 mM H_2_O_2_ treatment. As shown in Figure 3C, all four curcumin concentrations alleviated H_2_O_2_-induced cellular damage to varying degrees and improved cell viability. Notably, 20 μM curcumin effectively alleviated H_2_O_2_-induced cellular damage in both the N42V and A96T mutant strains.

The antioxidant stress protection conferred by curcumin to mutant strains was further evaluated using colony-forming unit (CFU) analysis (Figure 3D). In the CFU assay, Δ*sdh2(+)* and mutant strains exhibited varying degrees of sensitivity to H_2_O_2_ relative to their respective untreated controls (set as 100%). Notably, 20 μM curcumin-treated mutant strains under oxidative stress showed significantly improved viability. These findings further confirm the protective role of curcumin in yeast under oxidative stress conditions. Further validation based on the tablet phenotype, Compared to the Δ*sdh2(+)*, the mutant strain exhibited greater sensitivity to H_2_O_2_-induced oxidative stress. However, curcumin treatment mitigated the growth inhibition of the mutant strain under exposure to 1.5 mM H_2_O_2_.

Based on the above results of cell viability and plate phenotype assays, 20 μM curcumin exhibited a protective effect on SDH mutant strains under oxidative stress conditions by improving cell viability. In contrast, treatment with 50–200 μM curcumin alone exerted an inhibitory effect on the N42V mutant strain, leading to decreased cell viability. To further investigate the impact of different concentrations of curcumin on complex II functional defects, 20 μM and 200 μM were selected as the representative protective and inhibitory concentrations, respectively, for subsequent experiments.

### 2.4. Low-Concentration Curcumin Positively Regulates Mitochondrial Function Mitochondrial Function

**Figure 4:**
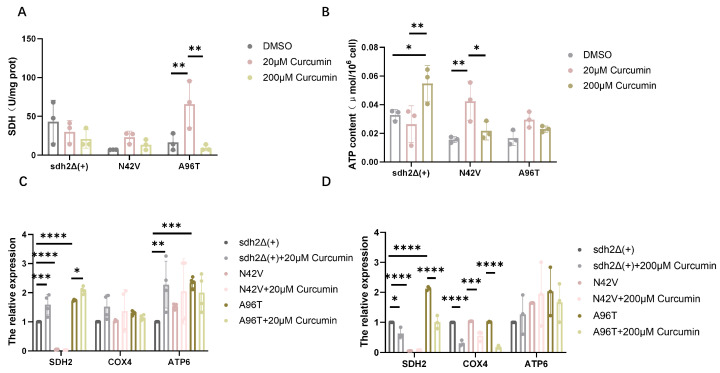
Low-concentration curcumin positively regulates mitochondrial function. (**A**) Effect of different concentrations of curcumin on SDH activity, normalized to total protein (U/mg protein); (**B**) Effect of different concentrations of curcumin on ATP content, normalized to cell number (hemocytometer) (μmol/$10^{6}$ cells); (**C**) Expression levels of respiratory chain-related genes detected by qRT-PCR under the influence of 20 μM curcumin; (**D**) Expression levels of respiratory chain-related genes detected by qRT-PCR under the influence of 200 μM curcumin. Data are presented as relative expression levels calculated using the $2^{-\Delta \Delta C_{t}}$ method (normalized to the control group, set as 1). All data are presented as mean ± SD from at least three independent experiments. For (**A**,**B**), one-way ANOVA was performed with curcumin concentration (0, 20, 200 μM) as the factor for each strain (Δ*sdh2(+)*, N42V, A96T). For (**C**,**D**), For each gene (*SDH2*, *COX4*, *ATP6*), two-way ANOVA was performed with treatment (control vs. curcumin) and strain (Δ*sdh2(+)*, N42V, A96T) as the two factors. Tukey’s post hoc test was used for multiple comparisons with correction. * *p* < 0.05, ** *p* < 0.01, *** *p* < 0.001, **** *p* < 0.0001.

To further investigate the effects of curcumin on mitochondria-related metabolism, we measured cellular energy levels and SDH activity (Figure 4A,B). Compared with the control group, treatment with 20 μM curcumin led to varying degrees of increase in both SDH activity and ATP content across the mutant strains, with the A96T mutant showing a particularly significant increase in SDH activity and the N42V mutant exhibiting a marked increase in ATP content. In contrast, treatment with 200 μM curcumin did not result in significant changes in either SDH activity or ATP content in the mutant strains. These findings indicate that 20 μM curcumin exerts a positive regulatory effect on mitochondrial function in the mutant strains, whereas 200 μM curcumin has no significant effect. Furthermore, the response to curcumin differs among the various mutation sites.

To evaluate the effects of curcumin on the transcriptional levels of genes involved in the mitochondrial respiratory chain, this study selected key genes representing different complexes of the respiratory chain for transcriptional analysis. *SDH2*, *COX4*, and *ATP6* correspond to respiratory chain Complex II, Complex IV, and Complex V, respectively, each located at distinct nodes of the mitochondrial oxidative phosphorylation pathway. Specifically, *SDH2* is involved in the coupling of the tricarboxylic acid cycle to electron transfer, *COX4* is a core component of the terminal oxidation step of the electron transport chain, and *ATP6* directly catalyzes ATP synthesis [24]. By examining the transcriptional changes in these three genes, the regulatory effects of curcumin on different steps of the oxidative phosphorylation pathway can be preliminarily assessed.

Compared with the control group, treatment with 20 μM curcumin significantly upregulated the transcriptional level of the *SDH2* gene in the Δ*sdh2(+)* strain and the A96T mutant strain (Figure 4C). In contrast, the *SDH2* transcript level in the N42V mutant strain was nearly undetectable, and curcumin treatment did not induce any observable change. Furthermore, the transcriptional levels of *COX4* and *ATP6* showed no significant changes in either mutant strain. When the curcumin concentration was increased to 200 μM (Figure 4D), the *SDH2* transcript levels in the Δ*sdh2(+)* strain and the A96T mutant strain were not elevated but were instead significantly downregulated. Meanwhile, the N42V mutant strain still exhibited no significant change, with *SDH2* transcript levels remaining near zero in both the treatment and control groups. Additionally, the transcriptional levels of *COX4* were significantly downregulated in all tested strains. In contrast, *ATP6* expression remained relatively stable across all strains, with no significant changes observed. Collectively, low-concentration curcumin partially upregulated the transcriptional levels of respiratory chain-related genes, whereas high-concentration curcumin exerted an inhibitory effect on the transcription of these genes.

### 2.5. Low-Concentration Curcumin Scavenges ROS in Cells

**Figure 5:**
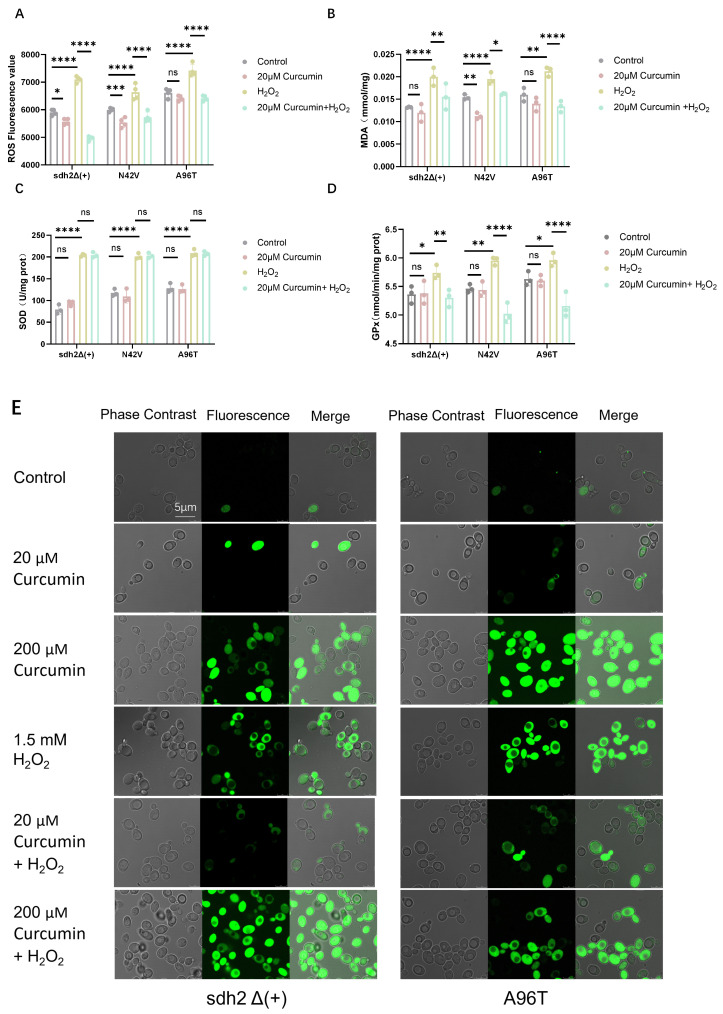
Low-concentration curcumin scavenges ROS in cells. (**A**) Detection of intracellular ROS levels under treatment with 20 μM curcumin and hydrogen peroxide (H_2_O_2_), Cell density was normalized to approximately $4\times 10^{6}$ cells per well (hemocytometer) to ensure comparable ROS detection conditions; (**B**) Detection of intracellular MDA levels under treatment with 20 μM curcumin and H_2_O_2_, normalized to total protein (nmol/mg protein); (**C**) Detection of intracellular SOD activity under treatment with 20 μM curcumin and H_2_O_2_, normalized to total protein (U/mg protein); (**D**) Detection of intracellular GPx activity under treatment with 20 μM curcumin and H_2_O_2_, normalized to total protein (nmol/min/mg protein). All data are presented as mean ± SD from three independent experiments. One-way ANOVA was performed with treatment (control, 20 μM curcumin, H_2_O_2_, 20 μM curcumin + H_2_O_2_) as the factor for each strain (Δ*sdh2(+)*, N42V, A96T), Tukey’s post hoc test was used for multiple comparisons with correction. ns, not significant, * *p* < 0.05, ** *p* < 0.01, *** *p* < 0.001, **** *p* < 0.0001. (**E**) Fluorescence imaging of intracellular ROS level detection under treatment with 200 μM curcumin and H_2_O_2_. Scale bars = 5 μm.

ROS serve as key indicators of oxidative stress in living organisms. To investigate the potential antioxidant role of curcumin in yeast cells, we quantified ROS levels in both stressed and non-stressed cells, with H_2_O_2_ employed as the oxidative stress inducer, in the presence or absence of curcumin in the culture medium. Both *sdh2*Δ(+) and mutant strains exhibited elevated ROS levels upon H_2_O_2_ treatment; however, ROS levels were significantly decreased in curcumin-treated stressed cells compared to those treated with H_2_O_2_ alone. This finding indicates that curcumin attenuates H_2_O_2_-induced intracellular oxidative stress and protects cells from H_2_O_2_-mediated damage (Figure 5A). Consistently, a higher proportion of green fluorescent cells was observed in H_2_O_2_-treated *sdh2*Δ(+) and A96T strains relative to their counterparts exposed to both H_2_O_2_ and curcumin, further demonstrating curcumin’s capacity to reduce intracellular oxidative stress (Figure 5E).

MDA is a natural byproduct of lipid peroxidation in organisms, and its quantification serves as an indicator of the extent of lipid oxidative damage. Both *sdh2*Δ(+) and mutant strains exhibited elevated MDA levels following hydrogen peroxide (H_2_O_2_) treatment (Figure 5B). Compared to the H_2_O_2_-treated group, MDA levels in curcumin-treated stressed cells were reduced to varying degrees, demonstrating that curcumin effectively attenuates H_2_O_2_-induced oxidative stress and inhibits lipid peroxidation.

SOD and GPx are key antioxidant enzymes that play vital roles in cellular defense against oxidative stress. SOD specifically catalyzes the dismutation reaction of superoxide anion radicals ($O_{2}^{-}$), converting them into hydrogen peroxide (H_2_O_2_) and oxygen, while GPx further reduces H_2_O_2_ to water using reduced glutathione (GSH) as a substrate, thereby protecting cells from ROS-induced damage. Treatment with H_2_O_2_ alone significantly induced the activities of SOD and GPx, indicating that the cells initiated an antioxidant defense response. In contrast, under oxidative stress conditions, curcumin-treated cells exhibited unchanged SOD activity (Figure 5C) and decreased GPx activity (Figure 5D) compared to the H_2_O_2_-alone group. These results suggest that low-concentration curcumin alleviates oxidative stress not through increasing the activities of these antioxidant enzymes.

### 2.6. High Concentrations of Curcumin Convert to Pro-Oxidants

**Figure 6:**
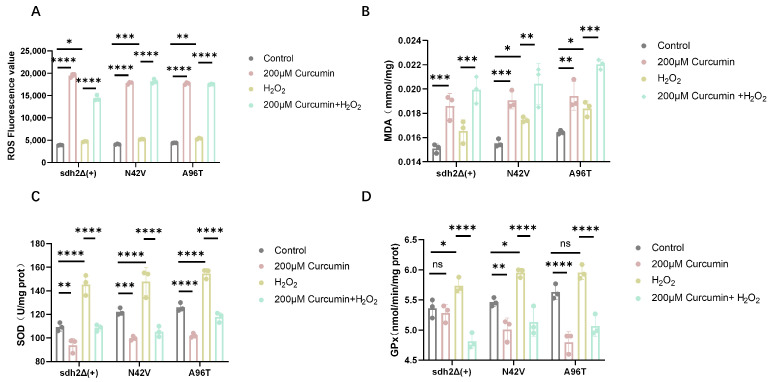
High concentrations of curcumin convert to pro-oxidants. (**A**) Detection of intracellular ROS levels under treatment with 200 μM curcumin and hydrogen peroxide (H_2_O_2_). Cell density was normalized to approximately $3\times 10^{6}$ cells per well (hemocytometer) to ensure comparable ROS detection conditions; (**B**) Detection of intracellular MDA levels under treatment with 200 μM curcumin and H_2_O_2_, normalized to total protein (nmol/mg protein); (**C**) Detection of intracellular SOD activity under treatment with 200 μM curcumin and H_2_O_2_, normalized to total protein (nmol/mg protein); (**D**) Detection of intracellular GPx activity under treatment with 200 μM curcumin and H_2_O_2_. All data are presented as mean ± SD from three independent experiments. One-way ANOVA was performed with treatment (control, 200 μM curcumin, H_2_O_2_, 200 μM curcumin + H_2_O_2_) as the factor for each strain (Δ*sdh2(+)*, N42V, A96T), Tukey’s post hoc test was used for multiple comparisons with correction. ns, not significant, * *p* < 0.05, ** *p* < 0.01, *** *p* < 0.001, **** *p* < 0.0001.

Under high-concentration curcumin treatment, intracellular ROS levels markedly increased in both stressed and non-stressed cells (Figure 6A), accompanied by a significant rise in the proportion of green fluorescent cells (Figure 5E), indicating enhanced oxidative stress. Concurrently, the change in MDA content exhibited a positive correlation with ROS levels, further confirming the exacerbation of oxidative damage, while the activities of SOD and GPx were both significantly reduced (Figure 6C,D). These findings indicate that high-concentration curcumin not only induces oxidative stress but may also suppress the endogenous antioxidant defense system, leading to decreased antioxidant enzyme activity.

## 3. Discussion

Although mitochondrial diseases are relatively rare, recent years have witnessed certain advances in their diagnosis and research [16]. However, due to the complexity of metabolic pathways and their regulation by genetic and environmental factors, therapeutic efficacy remains limited [25]. The lack of reliable model systems has further hindered the understanding of disease mechanisms and the development of therapeutic strategies [26]. In this study, using *Saccharomyces cerevisiae* as a platform, we introduced corresponding mutations to model complex II functional deficiency and employed hydrogen peroxide to simulate the state of exacerbated oxidative stress as seen in aged cells or late-stage disease. Our results show that curcumin ameliorates complex II deficiency by enhancing stress tolerance, restoring cell growth and viability, scavenging ROS, and positively regulates mitochondrial function, thereby providing experimental evidence for its potential application in the intervention of relevant human diseases.

It should be noted that the yeast model used in this study has certain limitations. The A96T mutation corresponds to the human Ala102 residue, which is fully conserved in *Saccharomyces cerevisiae* (Ala96); thus, this model can be directly used to simulate the human mutation. In contrast, the N42V mutation corresponds to the human Asp48 residue, which is not conserved in yeast (Asn42), and therefore cannot fully recapitulate all biochemical alterations of the human mutation. Nevertheless, Alston et al. (2012) [19] successfully validated the pathogenicity of the human SDHB p.Asp48Val mutation using the same yeast model, demonstrating its acceptable validity for mechanistic studies. The core conclusions of this study are primarily based on the conserved A96T model, while the results from the N42V model serve as supporting evidence that is consistent with the main findings, further enhancing the reliability of the conclusions.

Exposure of cells to low or high concentrations of curcumin respectively reduced or enhanced ROS production. The phenolic hydroxyl structure of curcumin confers strong antioxidant activity, enabling it to directly react with ROS [11]. In macrophage studies involving rat models, curcumin was shown to scavenge free radicals such as superoxide anions and hydroxyl radicals, and to effectively neutralize hydrogen peroxide [27]. In this study, treatment with H_2_O_2_ alone significantly induced the activities of SOD and GPx, indicating that the cells initiated an antioxidant defense response. Compared with the H_2_O_2_-alone group, stressed cells treated with low-concentration curcumin exhibited significantly reduced ROS levels, without a corresponding increase in SOD and GPx activities. The differential regulation of SOD and GPx by curcumin reveals its potential mechanism of action: curcumin does not inhibit H_2_O_2_-induced SOD elevation, suggesting that it does not interfere with the H_2_O_2_-mediated oxidative stress signaling pathway. In contrast, GPx activity was notably decreased in the presence of curcumin, implying that curcumin may directly scavenge H_2_O_2_, thereby reducing the cellular demand for GPx-mediated H_2_O_2_ clearance. This interpretation is consistent with the chemical properties of curcumin, as its phenolic hydroxyl groups can directly neutralize ROS, including H_2_O_2_. These results suggest that the antioxidant effect of curcumin is primarily achieved through direct scavenging of reactive oxygen species rather than through the induction of endogenous antioxidant enzyme systems.

In contrast to the antioxidant effects observed at low concentrations, high concentrations of curcumin may exhibit pro-oxidant properties [11]. Indeed, high concentrations of curcumin, as well as excessive exposure to environmental stressors such as ultraviolet radiation or blue light, may exert adverse effects on healthy tissues [28]. However, the precise mechanism by which high concentrations of curcumin induce excessive ROS production in yeast cells remains unclear. Multiple sources of intracellular ROS have been identified, including nitric oxide synthase, NADPH oxidase, fatty acid peroxisomes, xanthine oxidase, the mitochondrial electron transport chain, and endoplasmic reticulum (ER) stress [29]. Curcumin induces ER stress and ${\mathrm{Ca}}^{2+}$ release, leading to mitochondrial ROS production [30], As this pathway is conserved in yeast, we hypothesize that the elevated ROS following high-concentration curcumin treatment involves a similar mechanism. Validation requires future assessment of ER stress markers, ${\mathrm{Ca}}^{2+}$ levels, and IRE1/HAC1 pathway activity.

Curcumin has been reported to regulate mitochondrial function, alleviating dysfunction in diabetic and obese mice [31], and increasing ATP levels in yeast [12]. Although complex II deficiency severely impairs the mitochondrial respiratory chain, our findings show that curcumin improves mitochondrial metabolic phenotypes in mutant strains by enhancing SDH activity, ATP content, or respiratory gene transcription, thereby improving mitochondria-related biochemical parameters in the strains and exerting a positive regulatory effect on mitochondrial function in SDH2 mutants. It should be noted that this study did not verify Complex II assembly or enzymatic activity at the protein level, and the conclusions are primarily based on metabolic and transcriptional data. Therefore, the above conclusions warrant further validation at both the protein and respiratory function levels. Moreover, the nuclear-encoded *SDH2* and *COX4* were markedly affected by curcumin, whereas the mitochondrial-encoded *ATP6* was less responsive. Given that curcumin predominantly accumulates in the plasma membrane, nucleus, ER, and lysosomes, with minimal mitochondrial accumulation [32], this differential gene response may be attributed to the subcellular distribution of curcumin.

Although 200 μM curcumin increased total ATP levels in Δ*sdh2(+)* cells (Figure 4B), it did not upregulate *ATP6* transcription, indicating no enhancement of mitochondrial ATP synthesis. Instead, the elevated ATP likely reflects a compensatory response to high-concentration curcumin-induced oxidative stress and mild mitochondrial dysfunction, possibly involving alternative pathways such as glycolysis. Mutant strains, with pre-existing mitochondrial defects, lack sufficient compensatory capacity and thus show no ATP increase. The nearly undetectable *SDH2* transcript level in the N42V mutant is likely due to mRNA instability. The N42V mutation, located near the 5′UTR-CDS boundary, may disrupt local secondary structure, exposing degradation signals or nuclease-sensitive sites. Given that the SDH2 5′UTR confers intrinsic instability (half-life 5–7 min) [33], this mutation likely accelerates turnover, leading to near-zero steady-state levels.

The translation of these findings from yeast to humans has certain limitations. On the one hand, yeast is a unicellular organism that lacks the complex tissue–organ systems, drug metabolism processes, and regulatory networks present in humans. On the other hand, the curcumin concentrations used in this study (20–200 μM) are significantly higher than the plasma concentrations reported after oral administration (nanomolar to low micromolar range) [34]. Nevertheless, this study aims to elucidate the cellular mechanism of curcumin against complex II deficiency, rather than to mimic human pharmacokinetics. The cellular potential of curcumin revealed here does not depend on precise simulation of in vivo concentrations; however, whether effective concentrations can be achieved in humans remains a consideration for translational application.

Notably, poor bioavailability is a major bottleneck limiting the biological effects of curcumin, although recent advances are addressing this issue. Through structural modification and novel delivery systems, the druggability of curcumin has been significantly improved. Antony et al. reported that the proprietary formulation BCM-95^®^CG exhibited approximately 6.93-fold higher relative bioavailability than free curcumin, and 6.3-fold higher than a curcumin–lecithin–piperine formulation [35]. CurQfen (curcumin–fenugreek galactomannan complex) also enables efficient delivery of free curcumin in humans [36]. Although the concentrations used in this study are much higher than physiological levels, novel formulations are effectively addressing this bottleneck. Future studies should employ these high-bioavailability formulations or animal models to validate the in vivo protective effects of curcumin against complex II deficiency and to promote clinical translation.

## 4. Materials and Methods

### 4.1. Yeast Strain and Growth Conditions

*Saccharomyces cerevisiae* wild-type strain BY4741 was used in this study. Cells were cultured in YPD medium consisting of 1% yeast extract, 2% peptone and 2% glucose. The mutant strain was maintained on YPD agar plates supplemented with 200 μg/mL Geneticin for selection. For all experiments, cells were grown in YPD liquid medium in a shaking incubator at 30 °C and 200 rpm until reaching either the exponential or early stationary growth phase.

### 4.2. Curcumin Treatment

Curcumin (Beyotime, Shanghai, China) was dissolved in dimethyl sulfoxide (DMSO) to prepare a 100 mM stock solution, which was stored protected from light. Prior to use, the stock solution was diluted with YPD medium to the working concentrations. The final concentration of DMSO in all treatment groups was maintained below 0.1% (*v*/*v*) to avoid solvent toxicity. The control group received an equal volume of 0.1% DMSO as a vehicle control.

### 4.3. Mutant Strain Construction

Based on the *SDH2* gene sequence of *Saccharomyces cerevisiae* S288C, primers were designed to amplify the knockout cassette from the pUG6 plasmid. The *SDH2* gene was replaced by PCR-mediated homologous recombination using the geneticin resistance gene (*KanMX*) as a selectable marker, generating the BY4741Δ*sdh2* strain. Subsequently, the SDH2 fragment was amplified from the BY4741 genomic DNA and cloned into the pUC18 vector. Site-directed mutagenesis was introduced using a one-step cloning method to generate recombinant pUC18 plasmids harboring the desired mutations. After sequence verification, both the wild-type and mutant *SDH2* genes were subcloned into the pYX212 vector via restriction enzyme digestion and ligation, yielding the recombinant expression plasmids pYX212-SDH2 (wild-type) and pYX212-SDH2 (N42V/A96T). Finally, these recombinant plasmids were individually transformed into the BY4741Δ*sdh2* strain, and transformants were selected on SD-Ura plates. The resulting complemented strain was designated BY4741Δ*sdh2(+)*, and the mutant strains were designated BY4741-SDH2-N42V and BY4741-SDH2-A96T.

### 4.4. Sequencing Verification of Mutant Strains

To verify the successful construction of SDH2 mutant strains (N42V and A96T), recombinant pUC18 plasmids (carrying either the wild-type or mutant *SDH2* gene) were extracted from the corresponding strains. Sanger sequencing was performed using the universal primers M13F ($5^{\prime }$-TGTAAAACGACGGCCAGT-$3^{\prime }$) and M13R ($5^{\prime }$-CAGGAAACAGCTATGACC-$3^{\prime }$). Sequencing was conducted by Tsingke Biotechnology Co., Ltd. (Beijing, China). The resulting sequences were aligned with the wild-type SDH2 reference sequence to confirm the presence of the N42V and A96T mutations.

### 4.5. Growth Phenotypes of Yeast in Media with Different Carbon Sources

Single yeast colonies were picked and cultured overnight in YPD medium at 30 °C with shaking at 200 rpm. The cells were harvested, washed with 1× PBS, and resuspended to an OD600 of 1. The resulting suspension was then serially diluted 10-fold to concentrations of $10^{-1}$, $10^{-2}$, $10^{-3}$, $10^{-4}$, and $10^{-5}$. A 3 μL aliquot of each dilution was spotted onto YPD and YPG (1% yeast extract, 2% peptone, and 2% glycerol) solid media, respectively. The plates were incubated upside down at 30 °C for 3∼5 d, after which growth was monitored and recorded. YPG solid medium (1% yeast extract, 2% peptone, and 2% glycerol) was employed to evaluate strain growth on a non-fermentable carbon source (glycerol) medium [37]. 1× PBS consisted of 137 mM NaCl, 2.7 mM KCl, 10 mM Na_2_HPO_4_ and 1.8 mM KH_2_PO_4_ at pH 7.4.

### 4.6. Microcolony Counting

The test yeast strains were inoculated into YPD broth and cultured overnight. The cells were subsequently collected, washed three times with 1× PBS, and resuspended for OD600 measurement. The culture was then adjusted to an OD600 of 0.5 with 1× PBS [38], and 200 μL of the diluted suspension was spread onto YPD plates. Following incubation at 30 °C for 48 h [39], the proportion of microcolonies of the yeast strains was determined.

### 4.7. Determination of Cell Viability by CFU

Exponentially growing yeast strains were either treated with 1.5 mM H_2_O_2_ (Aladdin, Shanghai, China) and 20 μM curcumin for 8 h (the same duration as used in the MTT assay) or left untreated as controls. Serial 10-fold dilutions were prepared using 1× PBS to ensure that the final colony number was within the valid counting range of 30∼300 CFU per plate. The diluted yeast cultures were spread onto solid medium and incubated at a constant temperature. After incubation, plates containing 30∼300 colonies were selected for counting, and the average colony number of plates at the same dilution factor was calculated. The CFU value was then determined by multiplying this average by the corresponding dilution factor [40]. Viability was expressed as a percentage relative to the untreated control [41].

### 4.8. The Effect of Curcumin on Yeast Growth Phenotype Under Oxidative Stress

Logarithmic-phase cultures of both *sdh2*Δ(+) and mutant strains were either treated with curcumin or left untreated and incubated in a shaker for 2 h at 30 °C [40]. Following treatment, cells were serially diluted tenfold, and 3 μL aliquots of each dilution were spotted onto YPD agar plates supplemented with H_2_O_2_. The plates were incubated inverted at 30 °C for 3∼5 d, and yeast growth was subsequently monitored and recorded.

### 4.9. ROS Detection in Yeast Cells

ROS levels in yeast cells were detected using 2,7-dichlorofluorescein diacetate (DCFH-DA, Molecular Probes, Eugene, OR, USA). Exponentially growing yeast strains were either treated with curcumin and H_2_O_2_ or left untreated for 3 h. The treatment duration was based on previously reported protocols for yeast oxidative stress studies and was validated by preliminary experiments [40]. Following treatment, cells were washed with PBS and incubated with 20 μM DCFH-DA for 30 min at RT in the dark [42]. After incubation, cells were pelleted by centrifugation at 5000 rpm for 5 min and washed three times with PBS. ROS levels were then visualized by laser confocal microscopy and quantified using a fluorescence microplate reader (excitation/emission: 485/535 nm). All samples were measured using equal cell numbers (see figure legend for details), and the results were expressed as absolute fluorescence values.

### 4.10. Determination of Superoxide Dismutase Activity, Glutathione Peroxidase Activity and Lipid Peroxidation Level

Exponentially growing *sdh2*Δ(+) and mutant strains were treated with or without curcumin and H_2_O_2_ for 3 h, the same duration as used for the ROS assay. yeast cells were harvested into centrifuge tubes, the supernatant was discarded, and the cells were washed three times with 1× PBS (pH 7.4). Then, 5 × $10^{6}$ yeast cells were resuspended in 1 mL of extraction buffer, ultrasonically disrupted for 1 min, and centrifuged at 12,000× *g* for 10 min at 4 °C. The supernatant was collected for subsequent assays. Cellular lipid peroxidation levels and antioxidant enzyme activities were quantified using commercially available assay kits: malondialdehyde (MDA) assay kit for lipid peroxidation (Nanjing Jiancheng Bioengineering Institute A003, Nanjing, China), Total superoxide dismutase (SOD) assay kit (Nanjing Jiancheng Bioengineering Institute A001, Nanjing, China), and glutathione peroxidase (GPx) assay kit (Nanjing Jiancheng Bioengineering Institute A005, Nanjing, China) for enzyme activities, respectively. Activity was normalized to total protein.

### 4.11. MTT Assay

Exponentially growing *sdh2*Δ(+) and mutant strains were treated with different concentrations of H_2_O_2_ alone, curcumin alone, or H_2_O_2_ in combination with curcumin for 8 h (the 8-h treatment time was selected based on preliminary experiments, in which 4-h H_2_O_2_ treatment led to limited cell death, whereas 8-h treatment resulted in a stable and reproducible death phenotype), with untreated cells as controls. Cell viability was assessed using the MTT assay in yeast [43]. To address yeast cell wall permeability limitations on MTT entry, incubation time was optimized (30 min, 1 h, 2 h, 4 h, 6 h), with 4 h selected as optimal [21]. Cells were incubated with 20 μL of 5 mg/mL MTT solution for 4 h and centrifuged and the pellet was resuspended in DMSO to dissolve formazan crystals. Absorbance was measured at 490 nm.

### 4.12. Determination of SDH Activity and ATP Content

Exponentially growing *sdh2*Δ(+) and mutant strains were treated with or without curcumin for 3 h. This treatment duration was selected according to published literature on yeast mitochondrial dysfunction and was confirmed by preliminary experiments. Yeast cells were harvested into centrifuge tubes, the supernatant was discarded, and the cells were washed three times with 1× PBS (pH 7.4). According to the ratio of yeast cell number ($10^{4}$) to extract volume (mL) of 1000:1, the cells were ultrasonically disrupted for 1 min, then centrifuged at 11,000× *g* for 10 min at 4 °C. The supernatant was collected for subsequent assays. Cellular SDH activity and ATP content were quantified using the Succinate Dehydrogenase Assay Kit (Sangon^®^ Biotech D799375, Shanghai, China) and ATP Content Assay Kit (Yfxbio^®^ YFX0121, Nanjing, China), respectively. Activity was normalized to total protein or cell quantity.

### 4.13. Quantitative Real-Time PCR (qRT-PCR)

Yeast cells were lysed by grinding in liquid nitrogen, followed by total RNA extraction using RNAiso Plus (TaKaRa, Kusatsu, Japan) reagent. Complementary DNA (cDNA) was synthesized using the HiScript III RT SuperMix for qPCR (+gDNA wiper) Kit (Vazyme Biotechnology, Nanjing, China) [41]. Quantitative real-time PCR (qRT-PCR) was performed with Taq Pro Universal SYBR qPCR Master Mix (Vazyme Biotechnology, Nanjing, China) under the following thermal cycling conditions: initial denaturation at 95 °C for 10 min, followed by 35 cycles of 95 °C for 15 s and 60 °C for 1 min, and a final extension at 70 °C for 10 min. *ACT1* mRNA was used as the internal control, and relative gene expression levels were calculated using the $2^{-\Delta \Delta C_{t}}$ method. Supplementary Table S1 shows the primer sequences used for qRT-PCR.

### 4.14. Statistical Analysis

All experiments were performed independently at least three times (biological replicates), with three technical replicates per independent experiment. Data are presented as mean ± standard deviation (SD) from three independent experiments. For enzyme activity assays (SDH, SOD, GPx) and MDA content, raw values were normalized to total protein concentration and expressed as follows: SDH and SOD activities in U/mg protein, GPx activity in nmol/min/mg protein, and MDA content in nmol/mg protein. For ATP content, raw values were normalized to cell number (determined by hemocytometer counting) and expressed as nmol/$10^{6}$ cells. Comparisons between two groups were performed using two-tailed Student’s *t*-test. For multiple group comparisons, one-way or two-way analysis of variance (ANOVA) was used, followed by Tukey’s post hoc test. A *p*-value of <0.05 was considered statistically significant. All statistical analyses were conducted using GraphPad Prism version 8.0 software.

## 5. Conclusions

This study is the first to extend the yeast SDH mutation model from “pathogenic mutation validation” to “functional elucidation of natural products”, providing a low-cost platform for interventional studies on complex II deficiency. Low-concentration curcumin exerts its protective effects primarily through direct scavenging of ROS, while also positively regulating mitochondrial function, restoring cell growth and viability, and alleviating phenotypes associated with complex II dysfunction. In contrast, high-concentration curcumin promotes oxidative stress and downregulates the transcription of respiratory chain-related genes, thereby exacerbating mitochondrial dysfunction. Moreover, the concentration-dependent bidirectional regulatory effect of curcumin offers a reference for studying the dose–response relationship of natural products. It should be noted that this study is primarily based on phenotypic analyses using a yeast platform and did not rigorously validate the specific molecular mechanisms by which curcumin exerts its biological effects. Therefore, the above conclusions remain preliminary functional findings, and their translational applicability to higher organisms requires further validation.

## Data Availability

The original contributions presented in this study are included in the article. Further inquiries can be directed to the corresponding author.

## Supplementary Materials

The following supporting information can be downloaded at: https://www.mdpi.com/article/10.3390/ijms27125253/s1.

## Abbreviations

The following abbreviations are used in this manuscript:

MTT	3-[4-dimethylthiazol-2-yl]-2,5-diphenyltetrazolium bromide
Nrf-2	nuclear factor erythroid 2-related factor 2
Bcl-2	B-cell lymphoma 2
AP-1	Activator Protein 1
TORC1	Target of Rapamycin Complex 1
