# Supplementary material for: Curcumin Protects SDH2 Mutant from Oxidative Stress and Improves Mitochondrial Function: Application Potential for Complex II Deficiency
Source: Int J Mol Sci. 2026 Jun 10;27(12):5253. doi: 10.3390/ijms27125253 (PMC13299523; doi:10.3390/ijms27125253)
Supplement: Supplementary file 1 [file ijms-27-05253-s001.zip › ijms-4268046-supplementary.pdf]

Table S1 qRT-PCR primers

Primers	Sequence（5’→3’）
ACT1-F	GGTGATGGTGTTACTCACGTCG
ACT1-R	GTCAGTCAAATCTCTACCGGCC
SDH2-F	GTATGGCTACTGCCACAACAGC
SDH2-R	GCACTTGGCTCGTCTGGATT
COX4-F	GGTCCTGGTGCTAAAGAGGGTACC
COX4-R	TCATGGTACCCTTCCTGGACGAAT
ATP6-F	CGTACCTGCTGGTACACCATTACC
ATP6-R	GCCCAGACATATCCCTGAATGATACC
